# The Impact of Carbon on Electronic Structure of N-Doped ZnO Films: Scanning Photoelectron Microscopy Study and DFT Calculations

**DOI:** 10.3390/nano15010030

**Published:** 2024-12-27

**Authors:** Elzbieta Guziewicz, Sushma Mishra, Matteo Amati, Luca Gregoratti, Oksana Volnianska

**Affiliations:** 1 Institute of Physics, Polish Academy of Sciences, 02-668 Warsaw, Poland; shmbpj@ifpan.edu.pl; 2 Elettra-Sincrotrone Trieste S.C.p.A., SS 14-km Basovizza, 34149 Trieste, Italy; matteo.amati@elettra.eu (M.A.); luca.gregoratti@elettra.eu (L.G.)

**Keywords:** zinc oxide, p-type doping, photoelectron spectroscopy, Density Functional Theory calculations

## Abstract

A Scanning Photoelectron Microscopy (SPEM) experiment has been applied to ZnO:N films deposited by Atomic Layer Deposition (ALD) under O-rich conditions and post-growth annealed in oxygen at 800 °C. *State-of-the-Art* spatial resolution (130 nm) allows for probing the electronic structure of single column of growth. The samples were cleaved under ultra-high vacuum (UHV) conditions to open atomically clean cross-sectional areas for SPEM experiment. It has been shown that different columns reveal considerably different shape of the valence band (VB) photoemission spectra and that some of them are shifted towards the bandgap. The shift of the VB maximum, which is associated with hybridization with acceptor states, was found to be correlated with carbon content measured as a relative intensity of the C1s and Zn3d core levels. Generalized Gradient Approximation (GGA) supplemented by +U correction was applied to both Zn3d and O2p orbitals for calculation of the $V_{\mathrm{Zn}}$ migration properties by the Nudged Elastic Band (NEB) method. The results suggest that interstitial -${\mathrm{CH}}_{x}$ groups facilitate the formation of acceptor complexes due to additional lattice perturbation.

## 1. Introduction

The *p*-type conductivity of ZnO has been the subject of extensive research for over 20 years. Although the problem has not yet been solved, systematic studies combining modern research techniques supported by advanced theoretical calculations and tailored growth technology provide interesting new results. An important step forward was finding that the electron concentration of undoped ZnO layers is not determined solely by the native defects or hydrogen impurity, but by defect clusters containing both these components, such as $V_{\mathrm{Zn}}$H, $Zn_{i}V_{O}$H or *n*$V_{O}$, *n*$V_{\mathrm{Zn}}$, which was both predicted theoretically [1] and observed by many experimental techniques such as FTIR [2], Raman spectroscopy [3,4], and positron annihilation spectroscopy [5,6]. These defect clusters introduce deep and shallow levels in the bandgap, mostly donor-type, which explains a wide range of electron concentration values, which for undoped materials take values from $10^{14}$ to $10^{20}$/cm^3^ depending on growth conditions [7,8]. Consistently, *p*-type conductivity of ZnO is closely associated with defect clusters, which in addition to native defects ($V_{\mathrm{Zn}}$ in this case) also contain a dopant atom and sometimes hydrogen. For nitrogen, which is considered to be the best acceptor dopant in ZnO due to having an ionic radius and ionization energy similar to oxygen, defect clusters such as $V_{\mathrm{Zn}}$N, $V_{\mathrm{Zn}}$-$N_{O}$-$H_{x}$ or ($N_{O}$-$H_{x}$)*_Zn_* are considered [1,9]; these complexes may be formed in ZnO in many different ways depending on growth conditions. Because the formation of such clusters is much more complicated than the simple introduction of a substitutional dopant, the question arises as to whether such clusters can be formed in a controlled manner during the growth and/or post-growth processing of ZnO. The situation is made even more complex by the fact that the macroscopically measured *p*-type conductivity of ZnO may have a complicated microscopic origin, as shown by recent low temperature cathodoluminesence (LTCL) and scanning photoelectron spectroscopy (SPEM) studies that revealed separated donor and acceptor regions in ZnO:N [10,11,12]. Additionally, separated microregions with different conductivity type have been found in the surface photovoltage measurements of N-doped and N-As co-doped ZnO nanoparticles (NPs) [13,14].

Although the reasons for the formation of donor and acceptor domains in ZnO are not clear, it can be hypothesized that the distortion of the crystal lattice and the associated strain play a certain role. Defect complexes involve specific structural changes in the crystal lattice; thus, it can be assumed that appropriately introduced microstrain and surface proximity may facilitate their formation. Recent first-principle calculations indicate that the strain effect might be responsible for the formation of $V_{\mathrm{Zn}}$-H acceptor complexes, which in undoped ZnO can be formed in crystallites exhibiting compressive strains or near the surface [15]. In support of theoretical results, the low-temperature PL spectra measurements have shown considerably varying acceptor luminescence intensities for samples with different crystallographic orientation and strain [15].

In the present study we show that in addition to the strain/microstrain conditions inside the ZnO:N film, carbon incorporation may be an important factor facilitating the formation of acceptor complexes in ZnO. Similarly to hydrogen, carbon is a common impurity in ZnO that cannot be avoided in most growth procedures. The role of carbon has been already described in a series of theoretical and experimental works. Several groups have reported *p*-type conductivity for C-doped ZnO [16,17], while others have delivered contradictory results [18,19,20]. Based on Density Functional Theory (DFT) calculations, Sakong and Kratzer [21] considered charged carbon impurities in ZnO and found $C_{\mathrm{Zn}}$ to be the most stable configuration for a wide range of Fermi level positions. They found it to be a double donor for the lower $E_{F}$ position which converts to an acceptor for $E_{F}$ higher than 2.5 eV. However, these calculations yielded a ZnO bandgap of 1.65 eV, which challenges this finding. In turn, the DFT calculations of Lyons [22] et al. confirmed that $C_{\mathrm{Zn}}$ is the most stable configuration in ZnO and showed that it always acts as a double donor. In fact, recent research revealed the opening of the ZnO bandgap upon carbon doping and relative increase of electron concentration, indicating the occurrence the Burnstein–Moss effect [23]. However, it should be mentioned as well that there is experimental evidence showing that carbon can be passivated by hydrogen by forming the C-H complex, which is stable up to 1000 °C in the ZnO lattice [24]. On the other hand, there are also indications of stable C-N complexes with a low formation energy in ZnO doped with nitrogen [25,26]. It has been shown that the presence of carbon facilitates the incorporation of nitrogen, the amount of which in this case can reach up to several percent [26]. Additionally, DFT calculations of Liu et al. [27] demonstrate that the localized states of the C2p origin appear near the Fermi level, leading to the bandgap narrowing.

The SPEM investigations reported here show the modification of the valence band maximum (VBM) in ZnO doped with nitrogen, both *as-grown* and post-growth annealed. Photoemission spectra taken with *State-of-the-Art* resolution of 130 nm at different points of the ZnO:N film cross-section show the correlation of the VB shape and position with the carbon content. The low-intensity shape of the VBM and its shift towards the bandgap, which is a characteristic of acceptor domains [12], has been observed for crystallites with high carbon content.

According to a number of theoretical works [1,12], in the presence hydrogen, $V_{\mathrm{Zn}}$-$N_{O}$-$H_{x}$ is the dominant acceptor complex in ZnO:N, which introduces a deep acceptor state in the middle of the bandgap [1]. On the other hand, the $V_{\mathrm{Zn}}$-$N_{O}$ pair, which is a shallow acceptor with ionization energy of about 0.17 eV [9,12], could be created during the annealing process by removing hydrogen from the $V_{\mathrm{Zn}}$-$N_{O}$-$H_{x}$ complex [10,12]. Recent DFT calculations have revealed that the complexes involving zinc vacancy ($V_{\mathrm{Zn}}$) and nitrogen-substituting oxygen ($N_{O}$) modify the valence band maximum states; in case of the $V_{\mathrm{Zn}}$-$N_{O}$ pair, the VBM is moved towards the bandgap [12]. The DFT calculations presented here provide evidence that -CH and -${\mathrm{CH}}_{2}$ groups facilitate the formation of the above-mentioned acceptor defect complexes in the ZnO:N material.

## 2. Methods

### 2.1. Deposition of ZnO:N Thin Films

Nitrogen-doped zinc oxide films were grown on an Si(100) substrate by Atomic Layer Deposition (ALD) using a commercial Savannah-100 reactor from Cambridge Nanotech. The details of the growth process can be found elsewhere [11]. The films were deposited at 100 °C using a pulse time of 20 ms for both the deionized water and dimethylzinc precursors, while the nitrogen purging was set at 2 s. Such a low deposition temperature of the ALD process assures high oxygen content in the layer [8] and promotes the formation of zinc vacancy. Nitrogen was introduced into the films using an ammonia water (${\mathrm{NH}}_{4}$OH) precursor, alternating with a deionized water precursor every fourth ALD cycle, resulting in an N-doping level of 1·$10^{19}$at/cm^3^. ALD growth was performed for 10,000 cycles, resulting in a layer thickness of about 2.0 µm. Post-growth, Rapid Thermal Annealing (RTA) in oxygen atmosphere (800 °C, 3 min) was performed in an AccuTherm AW610 system from Alwin21 Inc. The goal of RTA was to remove excess hydrogen and allow the deep $V_{\mathrm{Zn}}$-$N_{O}$-H acceptor levels to be converted to shallow levels of $V_{\mathrm{Zn}}$-$N_{O}$ origin [10,11,12]. The annealing conditions were selected based on cathodoluminescence (CL) studies aimed at generating abundant acceptor centers in ZnO:N layers [10]. As previously shown, ZnO samples grown by ALD have high hydrogen content, which decreases after RTA to the level of $10^{19}$/cm^3^ [8,10]. The samples revealed a Wurtzite-type crystal structure and showed a polycrystalline nature with dominating 100 and 110 peaks, while transmission electron microscopy showed a columnar structure with ordered rows of atoms inside the grains [11].

### 2.2. Scanning Photoelectron Microscopy Measurements

**Figure 1:**
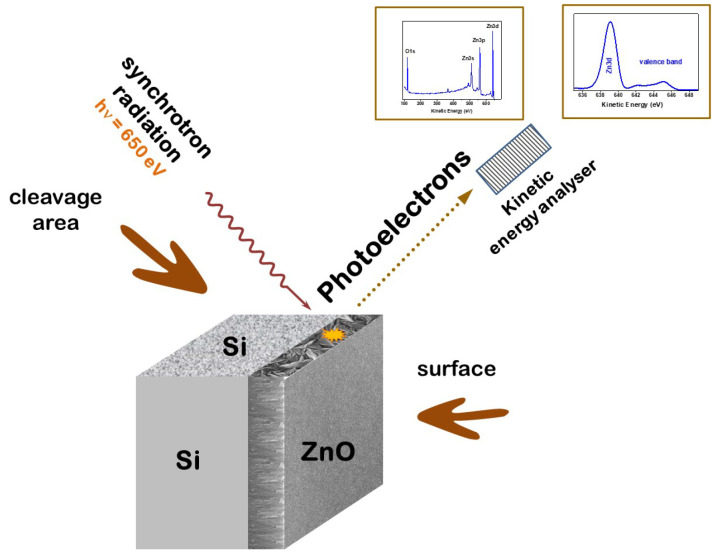
Geometry of the SPEM experiment.

The SPEM experiment was performed on the ESCA Microscopy beamline (Elettra, Trieste, Italy) with photon energy of 650 eV and energy resolution of 300 meV [28,29]. The spatial resolution of 130 nm, which is *State-of-the-Art* in scanning photoemission experiments, allowed for separate study of the electronic structure of each single growth column. The samples were cleaved inside a photoemission chamber under ultra-high vacuum (UHV) conditions, which allowed for obtaining an atomically clean cross-sectional area. The photoemission experiment was performed in situ immediately after cleavage. The experimental configuration is shown in Figure 1. The survey PES spectra were collected with an energy step of 0.656 eV and pass energy of 50 eV, while the valence band and the Zn3d and O1s core levels were recorded with an energy step 0.082 eV and pass energy of 20 eV. Before collecting the PES spectra, the Au foil in electrical contact with the investigated sample was measured and the binding energy was calibrated to the Au4f core level (84.0 eV), meaning that zero on the energy scale was related to the Fermi level. Additionally, in all presented spectra, the background was subtracted assuming a Shirley background. For comparison of spectra showing the valence band and the Zn3d core level, the intensity was normalized to 1 at the maximum of the Zn3d peak. The O1s peak was deconvoluted with a symmetric Gaussian function.

### 2.3. Computational Methods

Generalized Gradient Approximation (GGA) [30] was performed using the +U corrections [31] implemented in the QUANTUM-ESPRESSO package (QE) [32]. We employed ultrasoft atomic pseudopotentials [33] with valence orbitals of Zn(3$d^{10}$,4$s^{2}$) O (2$s^{2}$, 2$p^{4}$), N(2$s^{2}$, 2$p^{3}$), C(2$s^{2}$, 2$p^{2}$), and H(2$s^{2}$, 1$s^{1}$) and a plane wave kinetic energy cutoff of 40 Ry for wave functions. We used the Wurtzite 128-atom supercell and optimized atomic positions. For the supercell, the *k*-space summations were performed with a 2 × 2 × 2 k-points grid [34] or with a Γ-point. The +*U* terms of 10 eV and 7 eV were applied to the *d* (of Zn) and *p* (of O, N, and C) orbitals, respectively [12]. These values provide ZnO lattice parameters ($a_{0}$ = 3.223 Å and $c_{0}$ = 5.24 Å) that are consistent with the experimental data and the correct band structure of the material [12]. In particular, the calculated energy of forbidden gap was $E_{g}$ = 3.4 eV, which agrees well with the experimental results.

The defect migration properties were obtained by the Nudged Elastic Band (NEB) method implemented within the QE package [32].

The formation energies (*E*_form_) for O-rich conditions were calculated according to [35], and details are provided in [12]:

(1) $$E_{\mathrm{form}}=E_{\mathrm{tot}}\left(C_{i}H_{x}\text{:}\mathrm{ZnO}\right)-E_{\mathrm{tot}}\left(\mathrm{ZnO}\right)-\mu_{C}-x\mu_{H}$$

where $E_{\mathrm{tot}}$($C_{i}$$H_{x}$:ZnO) and $E_{\mathrm{tot}}$(ZnO) are the total energies of the supercell with and without the neutral $C_{i}$$H_{x}$, respectively, while $\mu_{C}$ and $\mu_{H}$ are the chemical potentials of C and H in the solid, and are referenced to the energy of bulk C (diamond structure) and per-atom energy of an isolated $H_{2}$ molecule, respectively.

## 3. Experimental Results

The cleavage area was investigated taking the imaging mode [28] of the ZnO:N/Si(100) film cross-section. We investigated both asgrown ZnO:N/Si film as well as the same film after 3 min of annealing in oxygen atmosphere at 800 °C. Such annealing conditions were found to be the most optimal for the activation of shallow acceptor states based on the cathodoluminescence studies of acceptor emission from ZnO:N films deposited in a similar ALD process [10].

**Figure 2:**
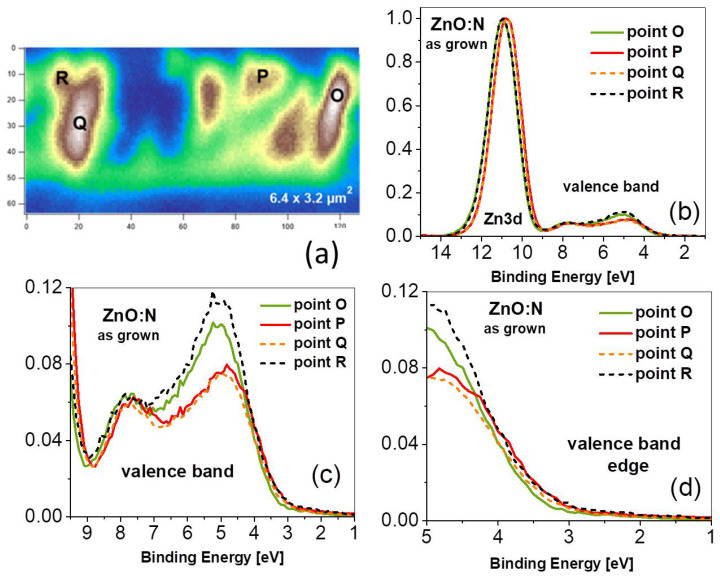
(**a**) SPEM image of the cross-section of the as-grown ZnO:N film (6.4×3.2 μm area), showing the points where photoemission spectra were taken; (**b**) photoemission spectra of the Zn3d and the valence band region taken at points O, P, Q, and R of the film cross-section; (**c**) the valence band region; and (**d**) the edge of the valence band.

Because the samples were mechanically broken inside the photoemission chamber, the cleavage area was not fully uniform and some parts of the cross-section were more or less exposed to the SPEM measurement (Figure 2a). As can be seen, the cross-sectional image of the ZnO:N layer is qualitatively similar to that obtained in a Scanning Electron Microscope (SEM) [36], and individual growth columns are easily distinguishable.

In the next step, the general photoemission spectra (PES), the valence band with the Zn3d core level, and the O1s and C1s spectra were measured at selected points of the cross-section area, as marked with black letters in Figure 2a. As shown in Figure S1 in the Supplementary Materials, only peaks characteristic of Zn, O, and sometimes C are visible in photoelectron spectra. The PES signal from the N1s core level was found below the detection limit because of the doping concentration (at the level of 10^19^/cm^3^ as measured by secondary ion mass spectrometry) and the extremely small sampling area.

The PES spectra of as-grown ZnO:N film measured in the valence band (VB) and the Zn3d core level region reveal a shift of the Zn3d core level and a different shape of the VB for measurements performed at different points of the film cross-section (Figure 2b). The spectra were taken in many different points of the ZnO:N film cross-section; for the clarity, four representative examples have been selected in Figure 2.

The valence-band spectrum of zinc oxide origins from the hybridized O2p and Zn4s electron shells consists of two maxima, one at about 5 eV and a second at about 7.7 eV below the Fermi level. The most substantial difference between PES spectra taken at different points of the film cross-section (i.e., for different growth columns) is the shape of the VB peak located at 5 eV below the Fermi level $E_{F}$ (see Figure 2b). The intensity of this maximum is about 30 percent higher for points O and R compared to points P and Q. A closer look at the VB edge (Figure 2d) shows that the spectra with lower intensity are slightly shifted towards the Fermi level, and that this shift can be evaluated to be less than 100 meV. It is accompanied by a shift in the same direction of the Zn3d core level, although the latter one is about twice as high. The full width at half-maximum (FWHM) of the Zn3d core level was found between 1.55–1.58 eV for the low-intensity spectra and at 1.62–1.67 eV for the high-intensity spectra. A shift of the core level towards lower binding energy can be understood in terms of a change in the Madelung energy, and is related to the shift of the VB positions towards $E_{F}$, which favors hybridization of the valence band with shallow acceptor states.

**Figure 3:**
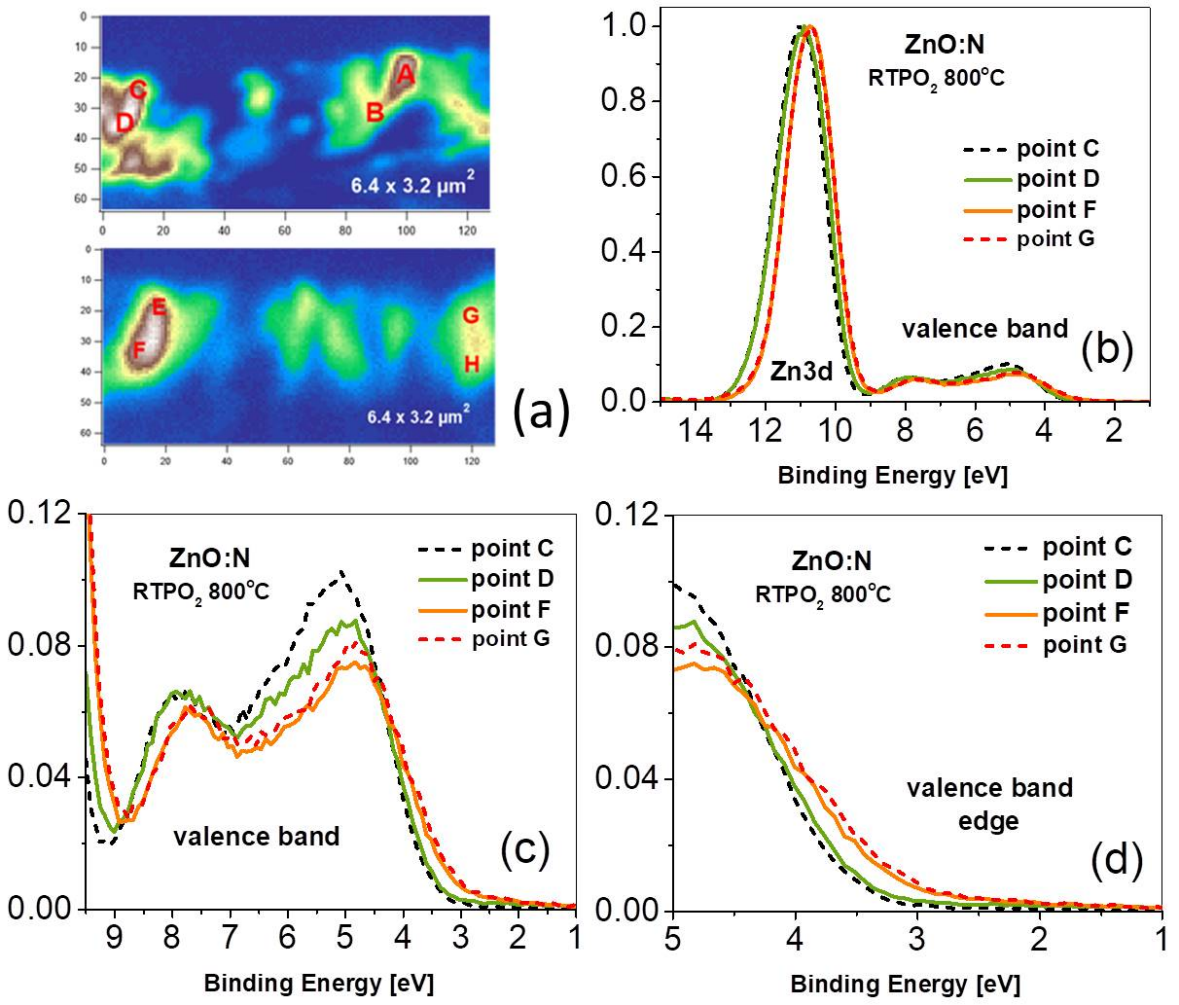
(**a**) SPEM image of the cross-section of the annealed ZnO:N film (6.4×3.2 μm area), showing the points where photoemission spectra were taken; (**b**) photoemission spectra of the Zn3d and the valence band region taken at points C, D, F, and G of the film cross-section; (**c**) the valence band region; and (**d**) the edge of the valence band.

The same set of results was obtained for the same ZnO:N sample but annealed at 800 °C in oxygen atmosphere (Figure 3). The cross-sections of the annealed ZnO:N film and selected points where PES spectra were taken are shown in Figure 3a. The PES spectra in the VB and the Zn3d binding energy region taken at points C, D, F, and G are shown in Figure 3b. The PES spectra were also taken in the rest of marked points; for clarity, we have chosen the most representative ones for comparison. Similar to the asgrown samples, after annealing we observe two kind of shapes of the PES spectra in the VB region and a shift of the Zn3d towards the Fermi level; however, the differences between the as-grown and annealed samples are significant. First, the highest maxima at 5 eV are about 20 percent lower for the annealed sample (compare Figure 2b and Figure 3b), while the height of the maximum at 7.7 eV remains untouched. Second, a pronounced shift of the low-intensity spectra towards the Fermi level can be clearly seen at points F and G (Figure 3d). This shift reaches a value of about 350 meV, and all measured VB spectra can be divided into two types with respect to this shift; the low-intensity spectra are shifted towards $E_{F}$, while the high-intensity spectra are situated at higher binding energy. The accompanying shift of the Zn3d level takes the same value of 350 meV. It is worth noting that in all cases the shift towards $E_{F}$ shows only the low-intensity spectra. Such a shift of the VB edge towards the bandgap indicates hybridization of the valence band electrons with shallow acceptor states. In this respect, the lowering intensity of the maxima close to the VB edge can be regarded as a fingerprint of the formation of acceptor complexes involving nitrogen, zinc vacancy, and possibly hydrogen. This finding is supported by the recently-published DFT calculations showing that $V_{\mathrm{Zn}}$-$N_{O}$ and $V_{\mathrm{Zn}}$-$N_{O}$-H complexes in ZnO:N can modify the density of states near the VB edge [12].

**Figure 4:**
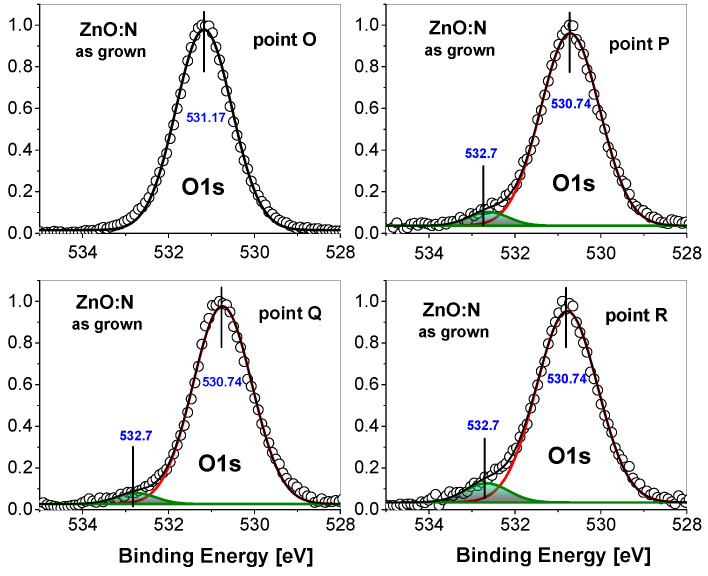
Photoemission spectra of the O1s core level in the as grown ZnO:N/Si film measured at points O, P, Q, and R of the film cross-section (points marked in Figure 2a).

A deeper insight into this issue can be gained by analyzing the O1s core level for as-grown and annealed samples. Deconvolution of the O1s peak measured for the as-grown ZnO:N film (Figure 4) shows only one contribution of this peak at point O and two of them at points P, Q, and R. At point O, the binding energy of the O1s level is 531.17 eV, while it is lower for the rest of the points, equaling 530.74 eV. The additional contribution visible at points P, Q, and R, is located at a binding energy about 1 eV higher. The main O1s peak comes from oxygen bound to zinc in the ZnO lattice. Comparison of the binding energy of the high-BE contribution with the reference book of standard photoemission spectra [37] and with previously published results for ZnO films obtained by ALD [8] indicates that it comes from oxygen bound to carbon. Because the ALD growth of ZnO layer uses the metal-organic precursor diethylzinc (DEZn), we can expect little carbon contamination. Indeed, carbon concentration as measured by Secondary Ion Mass Spectrometry (SIMS) was at the level of $1\times 10^{19}$ and $8\times 10^{18}$ at/cm^3^ for the as-grown and annealed films, respectively [10]. The binding energy of the C1s core level was measured as 284.2 eV (see Figure S2), which is characteristic of C-C bonding (configuration sp^3^) [38]. This confirms that carbon contamination derives from the DEZn precursor, which contains C_2_H_5_ chemical groups. As deionized water is also used in the ALD growth process, some contribution to the O1s peak from -OH groups cannot be excluded, especially because according to the reference book [37], the contribution from -OH groups can be expected in a similar BE range. However, comparing the present O1s spectra with the previously published ones [8], we see that the high-BE contributions shown in Figure 4 are relatively small; therefore, it is difficult to judge whether they consist of one peak or two.

**Figure 5:**
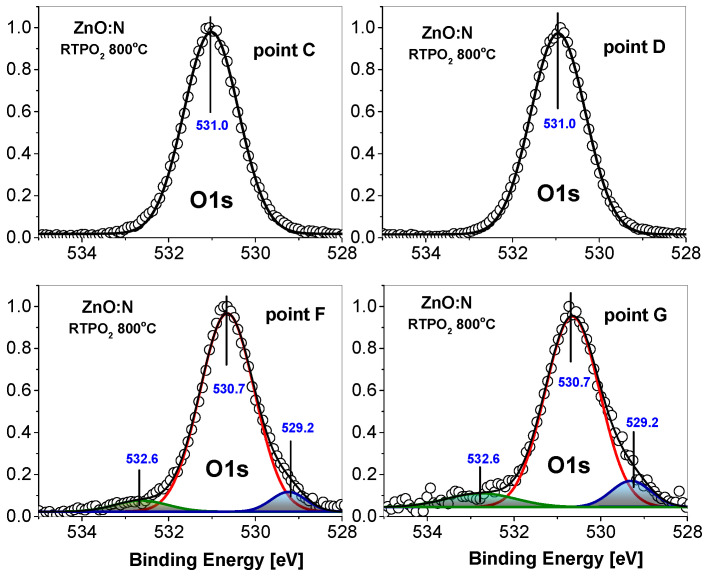
Photoemission spectra of the O1s core level in the annealed ZnO:N/Si film measured at points C, D, G, and F of the film cross-section (points marked in Figure 3a).

This small high-BE contribution is related to the way of performing the photoemission measurement, which was done in situ on an atomically clean surface created by cleaving the sample in vacuum. Typically, XPS spectra are taken from the surface, where there is an additional contribution from adsorbed CO_2_ molecules [39]. These molecules remain on the surface even after sputtering, and are visible in the photoemission spectra due to the extremely high surface sensitivity of this technique (sampling depth of 2–3 nm). In this sense, the O1s spectrum measured at point O are unique, similar to the spectra for points C and D presented in Figure 5, exclusively showing the signal from oxygen bound to the lattice zinc atoms with no additional contributions. These results have not been presented previously for ZnO films; in fact, for points C and D, the survey spectra do not show any carbon content (see Figure S1 in the Supplementary Materials).

A significant difference in the O1s spectra is visible after annealing (Figure 5). In this case, we observe two types of spectra. For measurements performed at points C and D, we see only a single O1s peak situated at BE of 531.0 eV, while at points F and G the BE of this peak is lower (530.65 eV) and two additional contributions are visible. The contribution at higher BE appears at 532.6 eV, which is very similar to the BE of this peak observed for as-grown sample. Moreover, the intensity of this peak compared to the main peak is also similar. This similar dependence of the contributions at higher binding energies for annealed and unannealed samples supports the assignment to the oxygen atoms bonded to carbon, as previously-published results clearly indicate the disappearance of -OH groups after annealing of ZnO layers in oxygen at 800 °C [40]. A very interesting effect is the appearance of the O1s contribution at lower BE of 529.3 eV, visible at points F and G. To the best of our knowledge, such a low-BE contribution has never been reported before for ZnO films. According to Moulder et al. [37], such a low BE of the O1s level is expected for oxygen atoms in metal oxides. Because the main O1s peak already comes from oxygen bound to metal (zinc) atoms in the ZnO lattice, we assumed another origin of this contribution, and tentatively assigned it to oxygen atoms in vicinity of acceptor complexes, which are expected to be formed in ZnO:N film grown in O-rich conditions and annealed in oxygen [12]. This hypothesis is supported by the valence band shift towards a bandgap that is observed in the photoemission spectra taken at the same F and G points (see Figure 3d). Interestingly, the O1s spectra collected at points C and D, located at higher BE of 531.0 eV, do not show any contributions to the main peak. Accordingly, the valence band spectrum measured at these points is also shifted towards higher BE (see Figure 3d).

The analysis of the valence band shift for all the examined cross-section points shows a strong correlation between the carbon content and the valence band shift towards the energy gap, which can be assigned to hybridization of valence band edge with shallow acceptor states. The theoretical calculations and discussion presented below aim to explain the role of carbon in the formation of acceptor states in nitrogen-doped ZnO.

## 4. Discussion

In our previous study [12], it was suggested from both experimental and theoretical points of view that the moderation of the valence band maximum (VBM) of ZnO:N and the VBM shift towards the bandgap is associated with the shallow acceptor states of the $V_{\mathrm{Zn}}$$N_{O}$ complexes. These complexes may be a product of dissociation reactions of $V_{\mathrm{Zn}}$$N_{O}$$H_{x}$ groups as a result of annealing processes, and could be associated with the expulsion of $H^{+}$ from the samples [10]. Moreover, the formation of the $V_{\mathrm{Zn}}$$N_{O}$$H_{x}$ groups depends on many factors of the deposition process; as shown, they are easily formed during the ALD growth with an ammonia water precursor [10]. The experimental results presented above show that unintentional carbon doping affects the shift of the PES spectra; therefore, we would like to understand the role of C impurity in this phenomena. This effect may have several different origins, such as the introduction of defect states in the vicinity of the VBM, or alternatively by favoring the formation of $V_{\mathrm{Zn}}$$N_{O}$ and/or $V_{\mathrm{Zn}}$$N_{O}$$H_{x}$ and/or C-$V_{\mathrm{Zn}}$$N_{O}$$H_{x}$ complexes or by introducing additional distortions into the crystal lattice [15], or finally by hydrogen absorption on the surface [41]. The last mechanism is unlikely to be the case in our experiments, as C ions already appear in the form of ${\mathrm{CH}}_{x}$ groups during the growth process [8]. There have been a number of important first-principles studies suggesting that C impurity in ZnO predominantly occurs via Zn ion substitution [21,22,42] or as interstitial lattice sites ($C_{i}$) [21,42], particularly under O-rich conditions, which is the case for our growth process. The $C_{Zn}$ ion can be regarded as a plausible candidate for a shallow donor dopant [22]. Moreover, the experiment in [23] showed that there is a gradual increase in optical $E_{g}$ with C doping, indicating the typical Burstein–Moss effect.

**Figure 6:**
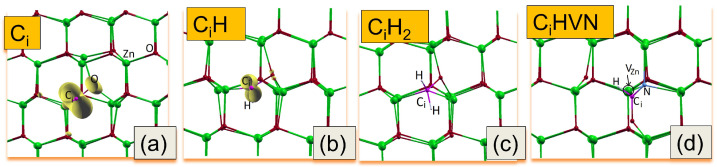
Equilibrium atomic configurations of the (**a**) $C_{i}$, (**b**) $C_{i}$-H, (**c**) $C_{i}$-$H_{2}$, and (**d**) $C_{i}$H–$V_{\mathrm{Zn}}$$N_{O}$ complexes, respectively. The yellow isosurfaces (corresponding to 0.01 electron/Bohr^3^) represent the electronic wave-density distributions. The Zn atoms are drawn with large spheres (green), the O, N, and C atoms with medium spheres (red, blue and purple), and the H atoms with small spheres (grey). The site of vacancy is depicted by the black circle.

Here, we have studied the $C_{i}$, $C_{i}$-$H_{x}$ (x = 1, 2) and $C_{i}$$H_{x}$–$V_{\mathrm{Zn}}$$N_{O}$$H_{x}$ complexes in terms of their structural stability, migration properties, and electronic properties. We note that the formation energies and computed Density of States (DOS) of $V_{\mathrm{Zn}}$$H_{x}$, $V_{\mathrm{Zn}}N_{O}H_{x}$ and $V_{\mathrm{Zn}}$$N_{O}$ complexes have been studied in our earlier works [12,15]. To find equilibrium configurations of defects, a few initial locations of atoms were checked. The obtained equilibrium configurations of $C_{i}$ and complexes are shown in Figure 6. In the equilibrium geometry, both $C_{i}$ and $C_{i}$-$H_{x}$ bind to neighboring O atoms, forming C-O complexes (see Figure 6a–c). This is reflected in their electronic wave distributions, depicted in Figure 6a,b by yellow color. The above C-O bonding states are also observed in core-level XPS spectra (Figure 4 and Figure 5). The gain $E_{\mathrm{gain}}$ in the formation energy of the complexes is evaluated as the difference in the formation energy between isolated constituents of the complex and the complex itself for a given Fermi level. A positive value of $E_{\mathrm{gain}}$ corresponds to a situation where there is an energetic preference for the defect complex to be formed. Our calculations have shown that these complexes are energetically stable by 3.2, 3.5, and 4.5 eV for $C_{i}$-H, $C_{i}$-$H_{2}$, and $C_{i}$H–$V_{\mathrm{Zn}}$$N_{O}$, respectively. We note that in the last case, that of $C_{i}$-H at an interstitial site, a defect complex engaging both a nearby Zn-vacancy and substituted nitrogen at O-site is formed. Complexes of the $C_{i}$-$V_{Zn}$ type in carbon-implanted ZnO nanowires were proposed in [43] as an origin of the observed magnetic properties. Thus, the formation of $C_{i}$H–$V_{\mathrm{Zn}}$$N_{O}$ may support the hypothesis that the presence of carbon–hydrogen groups may promote the clustering of acceptor complexes.

**Figure 7:**
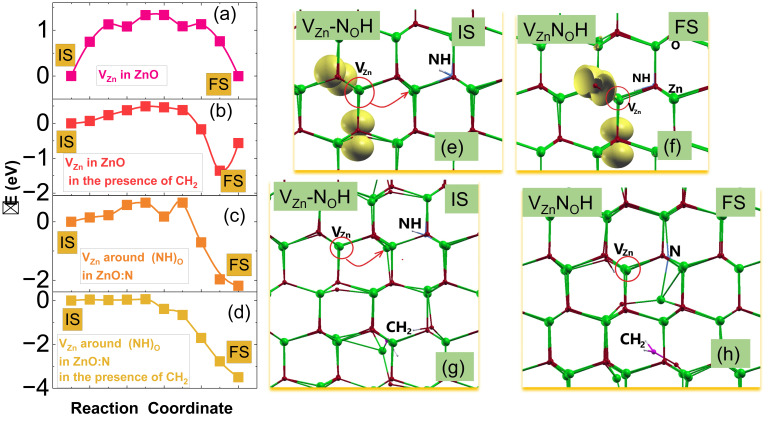
(**a**–**d**) Diffusion paths of the $V_{\mathrm{Zn}}$: (**a**,**b**) in ZnO without and with the presence of a ${\mathrm{CH}}_{2}$ group, respectively. (**c**,**d**) around the (NH)_O_ complex in N:ZnO without and with the presence of a ${\mathrm{CH}}_{2}$ group, respectively. (**e**–**h**) Atomic configurations of IS- or FS- states of $V_{\mathrm{Zn}}$ diffusion paths around (NH)_O_. The yellow isosurfaces (corresponding to 0.01 electron/Bohr^3^) represent the spin-density distributions for defect states. The complex is shown along *c*- axis. The site of the vacancy is depicted by the red circle, while the red arrows depict the migration direction.

Next, we studied the migration paths of zinc vacancy in ZnO and around the (NH)_O_ complex in N-doped ZnO under two conditions, namely, with and without the presence of ${\mathrm{CH}}_{2}$ groups in the samples. Using the NEB method, we calculated the diffusion paths for the exchange between zinc vacancy and its neighboring Zn atoms. Although there are two types of non-equilibrium paths, as one path is in the xy-plane and the other is along the c-axis, our calculations show that the two types of paths differ by less than 10 meV in undoped ZnO. Therefore, Figure 7 reports the results for the first path only, that is, the $V_{\mathrm{Zn}}$ migration from the initial state (IS) to the final state (FS). In Figure 7a–d, ΔE was calculated with respect to the energies of a system in the IS. Both the IS and FS for Zn vacancy migration paths around (NH)_O_ are shown in Figure 7e–h, both with and without the presence of ${\mathrm{CH}}_{2}$. Therefore, the FS corresponds to the equilibrium configuration of the $V_{\mathrm{Zn}}$$N_{O}$H complexes in both cases. The migration energy, i.e., the energy barrier $E_{m}$, is defined as the difference in energy between the IS and the energy of the so-called transition state (TS). The energy of the TS corresponds to the highest energy point of the lowest-energy migration path with respect to the IS energy, i.e., $E_{m}$ = E(TS) − E(IS). Moreover, the positive difference between IS and FS energies may indicate the energetic preference for the defect complex to be formed. We found that the $E_{m}$ of $V_{\mathrm{Zn}}$ in undoped ZnO is 1.33 eV, as displayed in Figure 7a, which is in reasonable agreement with other DFT results and experimental measurements [44]. Similarly, we calculated the diffusion path of $V_{\mathrm{Zn}}$ around(NH)_O_ in N-doped ZnO, finding that $V_{\mathrm{Zn}}$ can recombine with a neighboring (NH)_O_ group to form a $V_{\mathrm{Zn}}$$N_{O}$H complex by overcoming an $E_{m}$ of about 0.65 eV, as displayed in Figure 7c. Next, we studied the effect of the ${\mathrm{CH}}_{x}$ interstitial groups in the samples on the energy of migration barrier of $V_{\mathrm{Zn}}$ in both ZnO and N-doped ZnO. In this vein, we calculated vacancy migration paths affected by the presence of a ${\mathrm{CH}}_{2}$ group acting as interstitial impurities. We simulated the effect of local crystal deformations by randomly introducing a ${\mathrm{CH}}_{2}$ group separated from the vacancies by about 6 Å (see Figure 7g,h). Figure 7b,d presents these migration paths, showing that the migration barrier is reduced by 0.8 and 0.65 eV compared to the case without the carbon group. Moreover, we have shown that the difference between IS and FS energies increases; therefore, the acceptor complexes are more stable in the samples with carbon atoms. We point out that the energy barriers decrease significantly due to the increased atomic lattice disturbances generated by carbon groups. It has previously been suggested that microstrain/strain can affect the acceptor clustering of acceptors in ZnO [10,15] The effect of the lattice deformation and the localization of defect states on defect migration properties was shown in [45,46]. Moreover, the role of defect/vacancies migration in the formation of an additional phase or core-shell structures has been demonstrated in oxides as well as in Wurtzite nanocrystalline nitrides using the NEB and mesoscopic phase field methods [47,48]. Moreover, ref. [49] suggested within their bonding analysis that, as in the cation exchange process, the change of bonding energy can be dominated by the local lattice distortion contributions. We note as an example that the decreasing/increasing distance between O ions around the vacancy leads to increasing/decreasing electron–electron coupling. In particular, the outward displacement of O ions decreases the overlap between the broken bonds and consequently reduces the exchange interaction. Thus, it is clear from Figure 7e–h that the displacement of atoms around vacancies is stronger in structures with ${\mathrm{CH}}_{2}$ groups. Moreover, this atomic displacement not only extends to the nearest neighborhood, but also affects the farther neighbors.

In general, the formation of acceptor complexes such as $V_{\mathrm{Zn}}$ (NH)_O_ as a result of vacancy migration can be sensitive to the local atomic structure and perturbations. The introduction of even slightly asymmetric structures around vacancies can cause large changes in their symmetry structure, total energies, and electron states. We suggest that the mechanism of the formation of lowest-energy complexes may be driven by microstrain processes [50], including local lattice distortion provoked by unintentional impurities such as the ${\mathrm{CH}}_{x}$ groups in this work, external stress, or surface proximity, ultimately affecting the bonding environments and geometry of the defects [15]. In particular, in [15] we showed that both strain and surface proximity noticeably influence the formation energy of $V_{\mathrm{Zn}}$ in ZnO; therefore, acceptor states can be more easily formed in crystallites providing appropriate strain. In [15], we showed that photoluminescence spectra reveal different intensive acceptor luminescences for the ZnO layers with different crystallographic structures grown by Atomic Layer Deposition (ALD) on different substrates employing the same fabrication processes.

**Figure 8:**
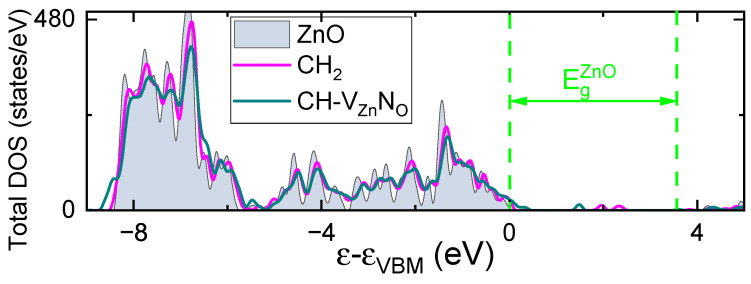
Total density of states of undoped ZnO and ZnO including ${\mathrm{CH}}_{2}$– and CH–$V_{\mathrm{Zn}}$$N_{O}$ complexes. The results are shown with respect to the VBM of undoped ZnO.

Finally, the calculated total DOS levels (see Figure 8) are in agreement with the photoelectron spectra results. In particular, we see a Zn3d core-level centered at about 8 eV below the VBM. This level is slightly shifted towards the VBM for dopants in ZnO. From the other side, the shift of the VBM into more higher energies take place in the $C_{i}$H-$V_{\mathrm{Zn}}$$N_{O}$ complex case. At the same time, we do not see this effect in the case of the $C_{i}$$H_{2}$ group. Note that the $C_{i}$$H_{2}$ interstitial introduces defect states in the middle part of the bandgap, and that these states are occupied by electrons; thus this complex is supposed to be a deep donor.

## 5. Summary

Photoelectron spectra of asgrown and annealed ZnO:N/Si films show significantly different intensities of the valence maximum close to the VB edge depending on the measured point of the film cross-section as well as a shift of some of these spectra towards the bandgap, pointing to hybridization with shallow acceptor states. We have found a significant correlation between a valence band shift and carbon content.

According to DFT calculations, $C_{i}$$H_{x}$ is introduced into the ZnO lattice as a stable interstitial group that forms C-H-O bond states. This group causes the nearby Zn vacancy to “sense” the -${\mathrm{NH}}_{O}$ group in N-doped ZnO. The calculated migration properties show that complexes such as $V_{\mathrm{Zn}}$(NH)_O_ are easily formed in the presence of the interstitial $C_{i}$$H_{2}$ group. In particular, we found that the migration barrier of the Zn vacancy is 1.33 and 0.65 in undoped ZnO and around (NH)_O_ in N:ZnO, respectively. The presence of ${\mathrm{CH}}_{2}$ groups in the samples leads to lowering of the migration energy by 0.8 eV and to zero in the ZnO and N:ZnO, respectively. We suggest that this may be due to stronger disorder of the crystal lattice. These results confirm that the formation of defect complexes is very sensitive to the local geometry and lattice perturbations.

## Data Availability

All data are contained within the article and Supplementary Materials. Further inquiries can be directed to the corresponding authors.

## Supplementary Materials

The following supporting information can be downloaded at: https://www.mdpi.com/article/10.3390/nano15010030/s1, Figure S1: Survey spectra taken at every measured points of the ZnO:N films cross-section; (left) as grown sample (points O, P, Q, R), (right) annealed sample (points C, D, F and G); Figure S2: Typical core level C1s spectrum.
