# Supplementary figures and images for: The Impact of Carbon on Electronic Structure of N-Doped ZnO Films: Scanning Photoelectron Microscopy Study and DFT Calculations
Source: Nanomaterials (Basel). 2024 Dec 27;15(1):30. doi: 10.3390/nano15010030 (PMC11723234; doi:10.3390/nano15010030)

## Supplementary Materials

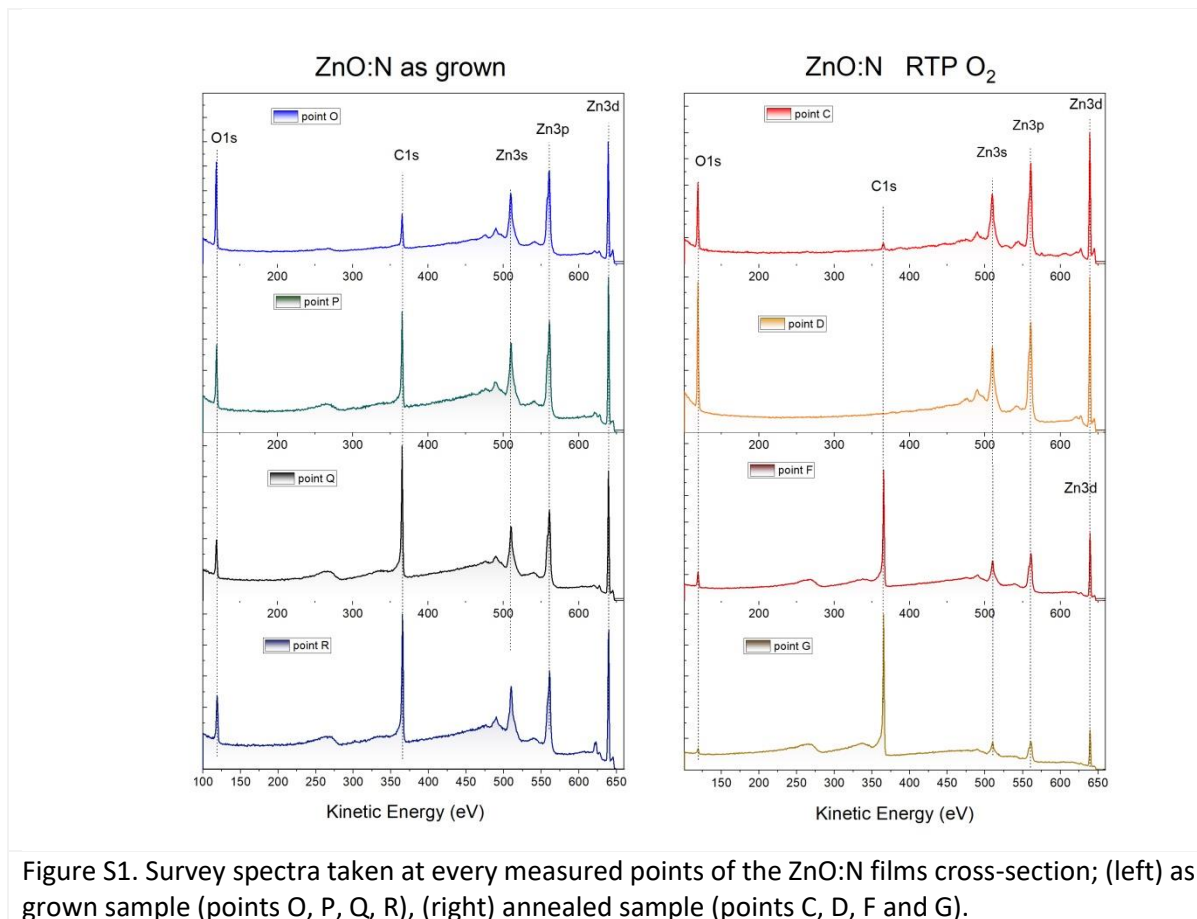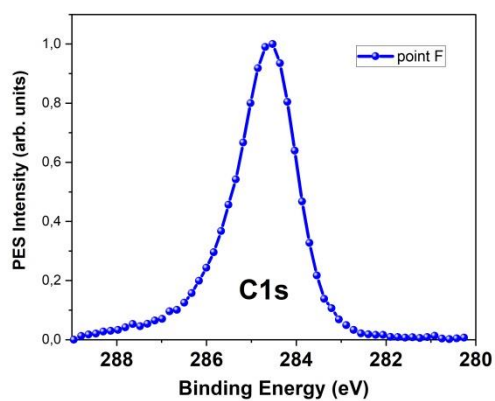

Supplement: Supplementary file 1 [file nanomaterials-15-00030-s001.zip › nanomaterials-3374573-supplementary.pdf]
